# Sex Difference in the Association Between Eating Away From Home and the Risk of High Serum Uric Acid in South China

**DOI:** 10.3389/fnut.2021.647287

**Published:** 2021-10-18

**Authors:** Shao-wei Chen, Ping Wang, Gui-yuan Ji, Qi Jiang, Xiao-min Hong, Wen-jun Ma, Rui Huang, Zi-hui Chen, Jie-wen Peng

**Affiliations:** Guangdong Provincial Center for Disease Control and Prevention, Guangdong Provincial Institute of Public Health, Guangzhou, China

**Keywords:** hyperuricemia, eating away from home, nutrition and health survey, dietary assessment, adult

## Abstract

**Background:** The prevalence of high serum uric acid is increasingly rising in recent years, and diet behavior is perceived to be associated with it. This study aimed to explore the relationship between eating away from home (EAFH) and the risk of high serum uric acid in adults in South China.

**Methods:** The data utilized in this study were from Guangdong Nutrition and Health Survey (NHS) 2015. Serum uric acid concentration was detected. EAFH in the past week was investigated. We defined EAFH as food consumption away from home. Dietary data were collected by 24-h recalls on 3 consecutive days. A generalized linear mixed-effects model was applied to compute the odds ratio (OR) and its corresponding 95% CI.

**Results:** A total of 3,489 individuals were included in this study. A 1.27-fold OR (95% CI: 1.05–1.52, *P* = 0.012) of high serum uric acid was identified in adults with EAFH in comparison with those without EAFH. With respect to men, a 1.66-fold OR (95% CI: 1.3–2.1, *P* < 0.001) of high serum uric acid was observed. We also observed that men with EAFH had higher intakes of red meat, poultry, vegetable, carbohydrate, protein, fat, and total energy, while a lower grain intake than those without EAFH. However, there was a lack of significant association between EAFH and the odds ratio of high serum uric acid in women. Women with EAFH did not have higher consumptions of red meat, vegetable, fish, fat, and water than those without EAFH.

**Conclusions:** This study found that EAFH was associated with an increased odds ratio of high serum uric acid in men, but not in women.

## Introduction

Hyperuricemia is perceived as a diet-related disease, which is characterized as the abnormal metabolism of purine (1). According to incomplete statistics, the global prevalence of hyperuricemia ranges from 6.7 to 17.1% (2). The prevalence of the disease in China was reported to be 13.3% (95% CI: 11.9–14.6%) (3), while it could be up to 25% in some coastal areas of the same country (3, 4), where citizens may have a high consumption of purine-rich foods such as seafood. Hyperuricemia could further develop to gout without implementation of any preventions and treatments. In addition, there is growing evidence that hyperuricemia is associated with an elevated risk of other diseases, such as hypertension, diabetes mellitus, and cardiovascular diseases (5–8).

Risk factors for hyperuricemia include age, sex, genetics, lifestyle, and environmental quality (9, 10). Previous publications have shown that diet patterns and behaviors exert an important role in the occurrence and development of hyperuricemia (11–13). Dietary sources are also considered as vital factors for diet health. Eating away from home (EAFH) was found to be associated with diet-related non-communicable diseases, such as obesity and diabetes (14–16). The plausible explanations could be a higher consumption of energy, fat, and meat, but lower consumption of minerals and vitamins such as calcium and vitamin A in individuals with EAFH (17, 18). However, some contrary views issued that eating at home may also adversely affect those who are responsible for preparing meals as they are more exposed to cooking fumes and other hazardous substances (19, 20). Therefore, the effect of EAFH on non-communicable diseases should be verified.

Guangdong province is located in South China and its economy has a rapid development since the reform and opening-up. Diet patterns and food sources in local citizens have dramatically changed in the last few decades. According to some recent studies, animal-derived foods account for an increasing proportion of the total caloric intake in this population (3, 21). Along with the improvement of living standards, the EAFH rate has been increased rapidly in Guangdong province in the past few years. However, whether NAFH has an impact on the occurrence and development of abnormally high serum uric acid has not been fully understood. Therefore, we conducted a cross-sectional study involving 3489 adult participants to explore it.

## Materials and Methods

### Study Design

**Figure 1:**
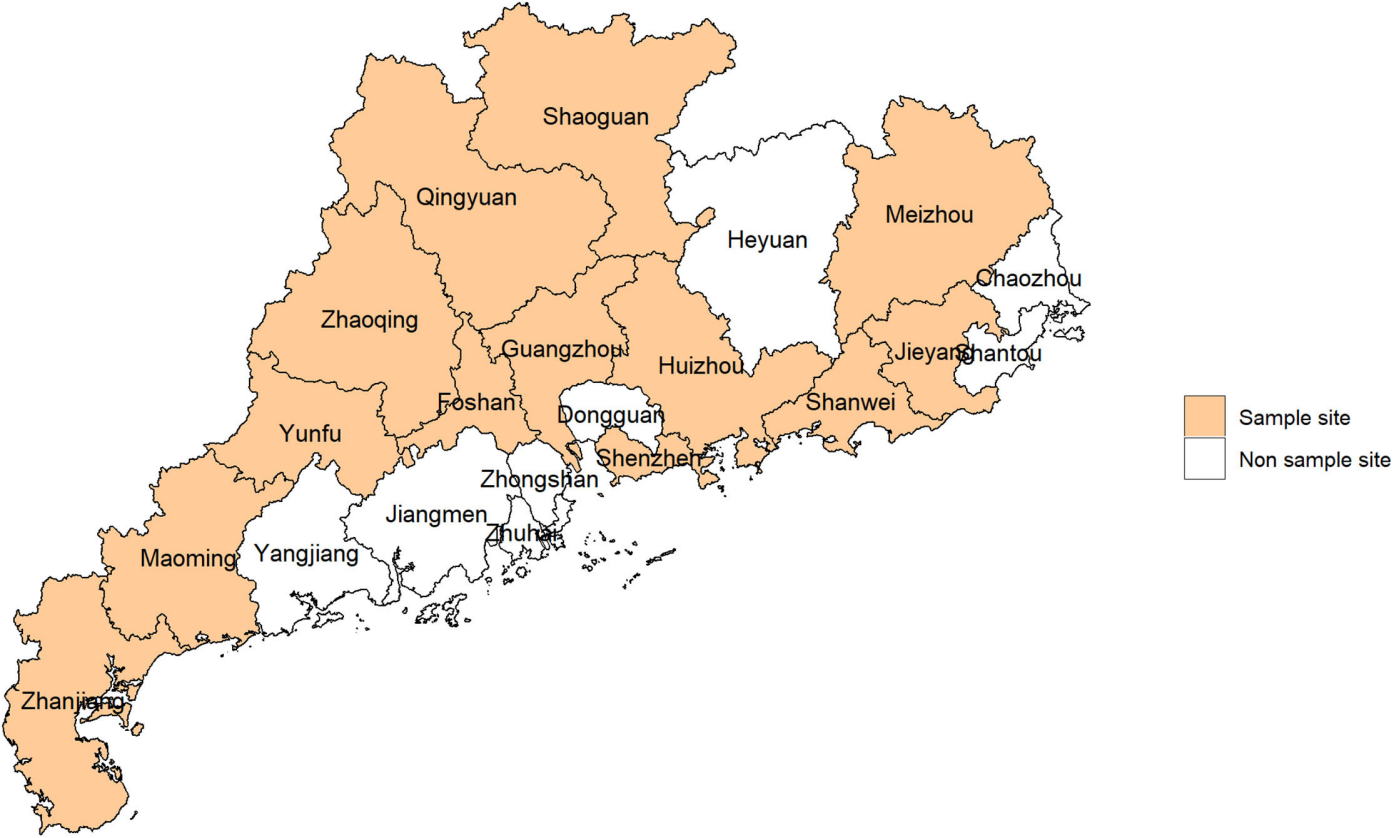
Sample sites in this study.

**Figure 2:**
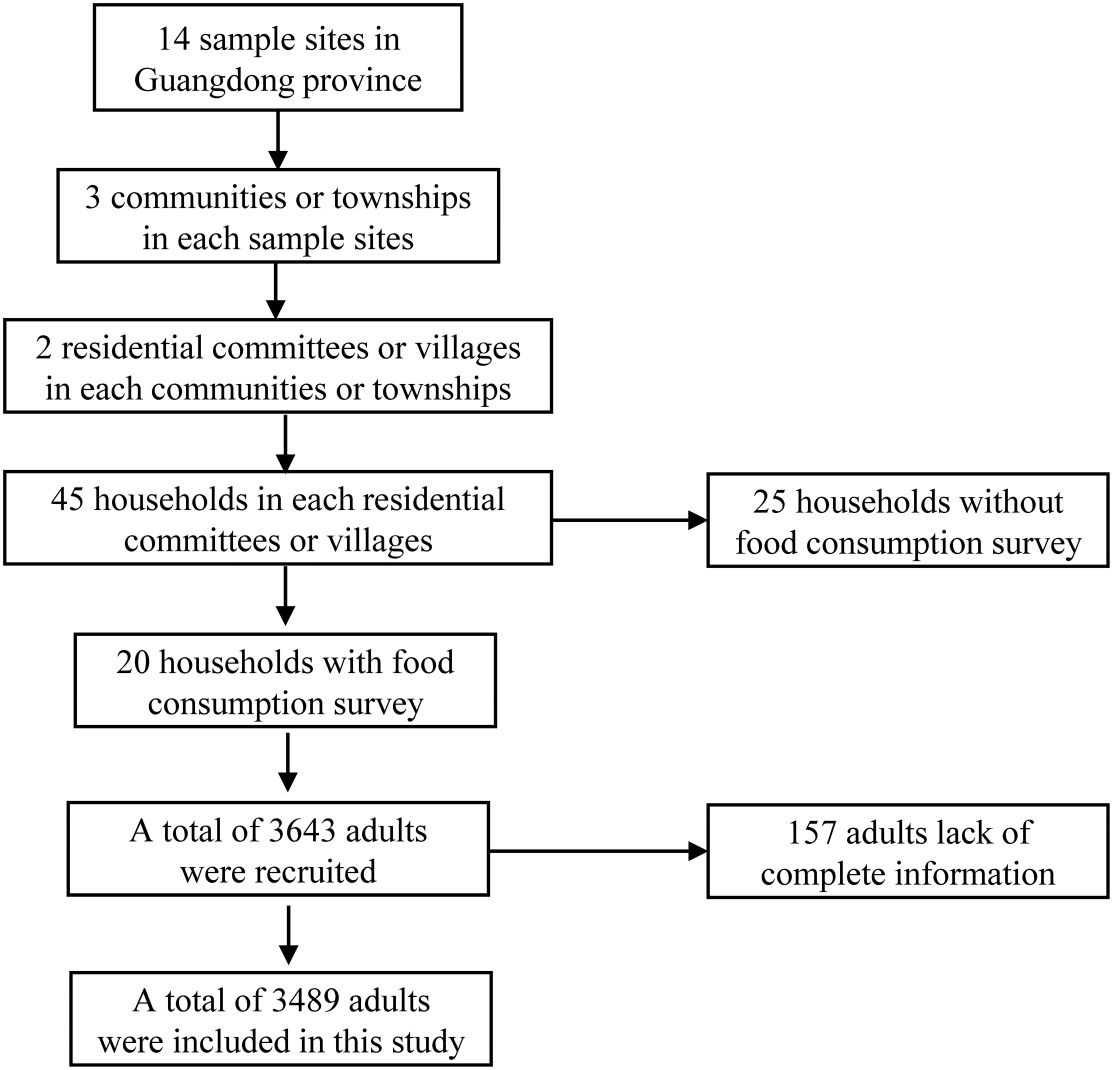
Flow chart of selecting participants.

Data utilized in this study were derived from Guangdong Nutrition and Health Survey (NHS) 2015, which was cross-sectional designed and a part of Chinese NHS 2015 (22). NHS was employed to evaluate food consumption, nutritional status, and their related factors. The study design was described in our previously published paper (23). Briefly, we applied a probability sampling method to select representative samples. There were 125 counties in Guangdong province, which were stratified into urban or rural layers and 14 counties were randomly selected (Figure 1). Afterward, three communities in the urban layer and three townships in the rural layer were further randomly selected. Finally, we randomly selected 45 households in each county, and individuals aged ≥18 years old in the households were recruited. Among the selected households, 20 households attended food consumption surveys and 25 households attended other health-related surveys. Participant selection is shown in Figure 2.

To obtain a minimum sample size in each county, the following formula was applied:


$$\begin{array}{l} N=deff\times \frac{u^{2}\times p\left(1-p\right)}{d^{2}} \end{array}$$

where N is the sample size, deff is design efficiency and set to be 3, u is the dividing value of normal distribution and set to be 1.96, p is probability and set to be 9.7% (the prevalence rate of diabetes mellitus in China in 2010), and d is deviation and set to be 0.0196 (20% × 9.7%). Thus, at least 612 adults were needed in each county.

A total of 8,991 adults were recruited in Guangdong NHS and 3,643 adults finished the food consumption survey. Since 157 participants lacked complete information, a total of 3,489 adults were included in this study.

### Dietary Data

Individual dietary data were collected by three consecutive 24-h recalls, 2 weekdays, and 1 weekend. Dietary assessment was performed by well-trained staff through face-to-face interviews and household surveys. All kinds of food and taking amount by participants in the 3 days were recorded. Food weight was evaluated by the investigator according to the recall of participants and with the help of a food map, comparison with the container, etc. Since cooking oil and condiments were consumed by family members at the household, we recorded their consumption amount at the household level. And then individual consumption of cooking oil and condiments was calculated based on the individual ratio of energy intake among family members and the times of taking meals at home. Major nutrients intake, including carbohydrate, protein, and fat, as well as total energy intake, were calculated according to the Chinese Food Composition Tables (2004 and 2009) (24, 25).

The dining places of participants in the past week were investigated. We defined EAFH as food consumption away from home in this study. In order to explore the combined effect of EAFH on high serum uric acid, adults who had more than one meal (breakfast, lunch, and dinner) EAFH (restaurant and/or company/school canteen) in the past week were perceived as having a habit of eating outside.

### Anthropometric and Biomarker Measurements

Anthropometric measurements were performed by well-trained investigators. Weight (kg), height (m), systolic blood pressure (mmHg), and diastolic blood pressure (mmHg) were measured, and their procedures were described elsewhere (23). Electronic bodyweight meter (HD-390, Tanita CO., LTD, China), electronic height meter (TZG, Wuxi Weighing Instrument CO., LTD, China), and electronic sphygmomanometer (HBP-1300, Omron CO., LTD, Japan) were used in measurements. Body mass index (BMI) was computed as weight (kg)/height (m^2^). Definition of hypertension was based on guidelines of the World Health Organization (WHO) (26).

The blood sample was collected from each participant in the morning after fasting for at least 8 h. The serum sample was separated after centrifugation. Serum uric acid concentration was determined by the oxidase method on an automated analyzer (Hitachi 7600, Hitachi Inc., Tokyo, Japan). Since serum uric acid was measured only once and serum sample from another day was no longer available, adults with hyperuricemia cannot be confirmed. Thus, we applied high serum uric acid to describe the abnormally high level of serum uric acid and it was defined as serum uric acid >420 μmol/L in men, while >360 μmol/L in women according to the definition of hyperuricemia in a previous study (27). Additionally, the participant with gout or with hyperuricemia treatment was also considered to be high serum uric acid.

### Statistical Analysis

Continuous variables were exhibited as mean and standard deviation if they were normal distribution, or exhibited as median and quartile if they were skewed distribution. Discrete variables were exhibited as number and proportion. To compare the differences of continuous variables (grain, vegetable, red meat, fish, poultry, water, carbohydrate, protein, fat, and total energy intake) between two groups (EAFH and non-EAFH), Student's *t*-test or Kruskal Wallis test was employed.

To explore the association between EAFH and the odds ratio of high serum uric acid, a generalized linear mixed-effects model was used and crude odds ratio (OR) and its corresponding 95% confidence interval (CI) were computed, as described in a previous study (23). We put possible confounding factors into the model to adjust the potential confounding effects and adjusted OR and its corresponding 95% CI were computed. Firstly, the effect of sex was adjusted. Secondly, the effect of age was adjusted. Thirdly, the effect of both sex and age was adjusted. Finally, the effect of multifactor, including sex, age, smoking, drinking, body mass index, sedentary leisure time, physical activity time, education, and marital status, was adjusted.

To obtain a robust association between EAFH and the odds ratio of high serum uric acid, subgroup analysis stratified by different sexes (men and women), meals (breakfast, lunch, and dinner), and dining out places (restaurant and canteen) were performed. Possible confounding effects mentioned previously were also adjusted applying different models.

Statistical analysis in the present study was performed in SAS Enterprise Guide (SAS Institute Inc., Cary, NC, USA) and R version 3.5.1 (R Core Development Team, Vienna, Austria). A *p*-value < 0.05 was characterized as statistically significant.

## Results

### Characteristics of Included Participants

A total of 3,489 adults with an average age of 52.0 ± 14.9 years were included in this study. Men accounted for 45.9% (1,603/3,489). The mean of serum uric acid was 342.4 ± 94.9 umol/L in the total sample, 385.9 ± 91.9 umol/L in men, and 305.3 ± 80.7 umol/L in women. The frequency of EAFH was 19.9% (696/3,489) in the total sample, 23.6% (378/1,603) in men, and 16.9 % (318/1,886) in women. The proportion of high serum uric acid was 26.4% (919/3,489) in the total sample, 32.5% (520/1,603) in men, and 21.2% (399/1,886) in women. Additional information about included participants is exhibited in Table 1.

**Table 1:** Characteristic of eligible participants.

**Variables**	**Male**	**Female**	**Overall**
	**(*n* = 1,603)**	**(*n* = 1,886)**	**(*n* = 3,489)**
Age (years)	52.9 ± 15.2	51.2 ± 14.7	52.0 ± 14.9
**Ethnicity (N, %)**			
Han	1,589 (99.4)	1,863 (99.0)	3,452 (99.2)
Other	9 (0.6)	18 (1.0)	27 (0.8)
BMI (Kg/m^2^)	23.3 ± 3.4	23.4 ± 3.6	23.4 ± 3.5
**Food intake (g/d)**			
Grain	237.7 (174.5, 313.5)	194.1 (146.2, 260.6)	215 (156.2, 283.9)
Vegetable	260.0 (190.0, 348.3)	258.3 (183.3, 343.3)	258.7 (186.7, 346.7)
Red meat	115.0 (66.7, 170)	90.0 (51.7, 137.3)	100.0 (60.0, 152.7)
Fish	40.0 (0, 91.8)	33.3 (0, 75)	35.1 (0, 82.9)
Poultry	19.7 (0, 53.3)	16.7 (0, 44)	17.6 (0, 48.7)
Water	751.2 (578.6, 958.5)	682.9 (528.6, 866.6)	713.8 (550.2, 913.1)
**Nutrient intake (g/d)**			
Carbohydrate	209.4 (162.8, 265.7)	182.7 (145.4, 230.5)	193.3 (152.2, 247.5)
Protein	67.4 (53.5, 83.3)	57.7 (46, 71.4)	61.7 (48.7, 77.5)
Fat	82.0 (61.1, 109.6)	69.2 (51.5, 91.9)	74.9 (54.9, 99.9)
Total energy intake (Kcal/d)	1899.0 (1538.5, 2316.2)	1614.2 (1323.4, 1941.4)	1739.3 (1398.7, 2130.5)
Sedentary leisure time (h/d)	5.1 ± 2.8	4.8 ± 2.8	4.9 ± 2.8
Physical activity time (h/d)	0.14 ± 0.47	0.05 ± 0.28	0.09 ± 0.38
Serum uric acid (umol/L)	385.9 ± 91.9	305.3 ± 80.7	342.4 ± 94.9
**Eating away from home (N, %)**			
Total	378 (23.6)	318 (16.9)	696 (19.9)
Breakfast	206 (12.9)	171 (9.1)	377 (10.8)
Lunch	260 (16.2)	229 (12.1)	489 (14.0)
Dinner	11.9 (7.4)	89 (4.7)	208 (6.0)
*Restaurant*	276 (17.2)	183 (9.7)	459 (13.2)
Canteen	147 (9.2)	166 (8.8)	313 (9.0)
High serum uric acid (N, %)	520 (32.5)	399 (21.2)	919 (26.4)
Hypertension (N, %)	522 (32.7)	545 (29.0)	1,067 (30.7)
Smoking (N, %)	896 (56.1)	46 (2.5)	942 (27.1)
Drinking (N, %)	852 (53.3)	470 (25.0)	1,322 (38.0)
**Education (N, %)**			
≤ 6 years	823 (51.5)	647 (34.4)	1,470 (42.3)
7–12 years	609 (38.1)	1,055 (56.1)	1,664 (47.8)
≥13 years	166 (10.4)	179 (9.5)	345 (9.9)
**Marital status (N, %)**			
Married	1,452 (90.9)	1,705 (90.6)	3,157 (90.7)
Other	146 (9.1)	176 (9.4)	322 (9.3)

*BMI, body mass index*.

### Association Between EAFH and the Odds Ratio of High Serum Uric Acid

A 1.27-fold OR (95%CI: 1.05–1.52, *P* = 0.012) of high serum uric acid was found in participants who had a habit of EAFH. The positive association remains after adjusting for age (OR: 1.34, 95% CI: 1.11–1.61, *P* = 0.002) or age and sex (OR: 1.25, 95% CI: 1.04–1.52, *P* = 0.019), while the association was not statistically significant after adjusting for sex (OR: 1.2, 95% CI: 0.99–1.44, *P* = 0.058) or multifactor (OR: 1.15, 95% CI: 0.93–1.41, *P* = 0.195). Detail results are shown in Table 2.

**Table 2:** Association between eating away from home and risk of high serum uric acid.

**Groups**	**Models**	**OR**	**95%CI**	***P*-value**
Total	Crude effect	1.27	1.05–1.52	0.012
	Adjusted for sex	1.20	0.99–1.44	0.058
	Adjusted for age	1.34	1.11–1.61	0.002
	Adjusted for sex and age	1.25	1.04–1.52	0.019
	Adjusted for multifactor^a^	1.15	0.93–1.41	0.195
Male	Crude effect	1.66	1.30–2.10	<0.001
	Adjusted for age	1.63	1.28–2.08	<0.001
	Adjusted for multifactor^b^	1.38	1.06–1.81	0.017
Female	Crude effect	0.72	0.53–0.99	0.044
	Adjusted for age	0.83	0.60–1.15	0.259
	Adjusted for multifactor^b^	0.89	0.63–1.26	0.514

a *Generalized linear mixed-effects model adjusted for sex, age, smoking, drinking, body mass index, sedentary leisure time, physical activity time, education, and marital status*;

b *Generalized linear mixed-effects model adjusted for age, smoking, drinking, body mass index, sedentary leisure time, physical activity time, education, and marital status*.

### Subgroup Analysis

#### Different Sexes

With respect to men, EAFH was found to be associated with an elevated OR of high serum uric acid applying a crude effect model (OR: 1.66, 95% CI: 1.3–2.1, *P* < 0.001), adjusting for age (OR: 1.63, 95% CI: 1.28–2.08, *P* < 0.001), or adjusting for multifactor (OR: 1.38, 95% CI: 1.06–1.81, *P* = 0.017). However, as for women, the associations were not statistically significant (Table 2).

#### Different Meals

Eating away from home during breakfast was found to be associated with an elevated OR of high serum uric acid whether applying a crude effect model or applying adjusted effect models. However, EAFH during lunch and dinner was not associated with the OR of high serum uric acid (Table 3). The disparity of the association between the two sexes was also observed regarding various meals.

**Table 3:** Association between eating away from home and risk of high serum uric acid regarding breakfast, lunch, and dinner.

**Groups**	**Models**	**Breakfast**			**Lunch**			**Dinner**		
		**OR**	**95% CI**	***P*-value**	**OR**	**95% CI**	***P*-value**	**OR**	**95% CI**	***P*-value**
Total	Crude effect	1.50	1.20–1.89	0.001	1.07	0.87–1.33	0.513	1.20	0.88–1.63	0.251
	Adjusted for sex	1.43	1.14–1.80	0.002	1.02	0.82–1.27	0.831	1.12	0.82–1.53	0.473
	Adjusted for age	1.56	1.24–1.97	<0.001	1.13	0.81–1.41	0.262	1.25	0.92–1.70	0.161
	Adjusted for sex and age	1.48	1.17–1.87	0.001	1.07	0.86–1.34	0.535	1.16	0.85–1.59	0.348
	Adjusted for multifactor^a^	1.37	1.07–1.75	0.012	0.94	0.74–1.19	0.593	1.04	0.75–1.45	0.805
Male	Crude effect	1.78	1.32–2.40	<0.001	1.54	1.17–2.02	0.002	1.56	1.07–2.28	0.022
	Adjusted for age	1.75	1.30–2.36	<0.001	1.50	1.14–1.99	0.004	1.52	1.04–2.23	0.031
	Adjusted for multifactor^b^	1.52	1.10–2.08	0.010	1.22	0.90–1.65	0.197	1.22	0.81–1.84	0.332
Female	Crude effect	1.03	0.70–1.51	0.886	0.50	0.33–0.75	0.001	0.57	0.30–1.05	0.071
	Adjusted for age	1.17	0.79–1.72	0.435	0.58	0.38–0.88	0.010	0.65	0.35–1.21	0.173
	Adjusted for multifactor^b^	1.23	0.82–1.85	0.321	0.61	0.39–0.94	0.027	0.72	0.38–1.37	0.317

a *Generalized linear mixed–effects model adjusted for sex, age, smoking, drinking, body mass index, sedentary leisure time, physical activity time, education, and marital status*;

b *Generalized linear mixed-effects model adjusted for age, smoking, drinking, body mass index, sedentary leisure time, physical activity time, education, and marital status*.

#### Different Dining Out Places

An increased OR of high serum uric acid was observed in EAFH at a restaurant, while EAFH in a canteen was not associated with the OR of high serum uric acid. Similar results about sex differences were also identified. Detailed results are summarized in Table 4.

**Table 4:** Association between eating away from home and risk of high serum uric acid regarding restaurant and canteen.

**Groups**	**Models**	**Restaurant**			**Canteen**		
		**OR**	**95% CI**	***P*-value**	**OR**	**95% CI**	***P*-value**
Total	Crude effect	1.51	1.22–1.83	<0.001	1.03	0.79–1.33	0.853
	Adjusted for sex	1.38	1.12–1.71	0.003	1.02	0.78–1.33	0.891
	Adjusted for age	1.57	1.28–1.95	<0.001	1.08	0.83–1.41	0.572
	Adjusted for sex and age	1.44	1.16–1.79	0.001	1.07	0.82–1.40	0.639
	Adjusted for multifactor^a^	1.33	1.06–1.68	0.013	0.95	0.71–1.26	0.706
Male	Crude effect	1.78	1.36–2.32	<0.001	1.49	1.05–2.11	0.025
	Adjusted for age	1.75	1.36–2.28	<0.001	1.45	1.02–2.05	0.040
	Adjusted for multifactor^b^	1.53	1.15–2.03	0.004	1.14	0.78–1.66	0.485
Female	Crude effect	0.87	0.59–1.28	0.469	0.60	0.39–0.95	0.027
	Adjusted for age	0.98	0.66–1.45	0.929	0.71	0.45–1.12	0.141
	Adjusted for multifactor^b^	1.08	0.71–1.63	0.733	0.76	0.47–1.22	0.250

a *Generalized linear mixed-effects model adjusted for sex, age, smoking, drinking, body mass index, sedentary leisure time, physical activity time, education, and marital status*;

b *Generalized linear mixed-effects model adjusted for age, smoking, drinking, body mass index, sedentary leisure time, physical activity time, education, and marital status*.

#### Food, Nutrients, and Energy Intake

Participants with EAFH consumed more red meat, poultry, fish, and vegetable than those with non-EAFH, while grain intake was the opposite. Men participants with EAFH had a higher intake of red meat, poultry, and vegetable, but a lower intake of grain than those with non-EAFH.

**Figure 3:**
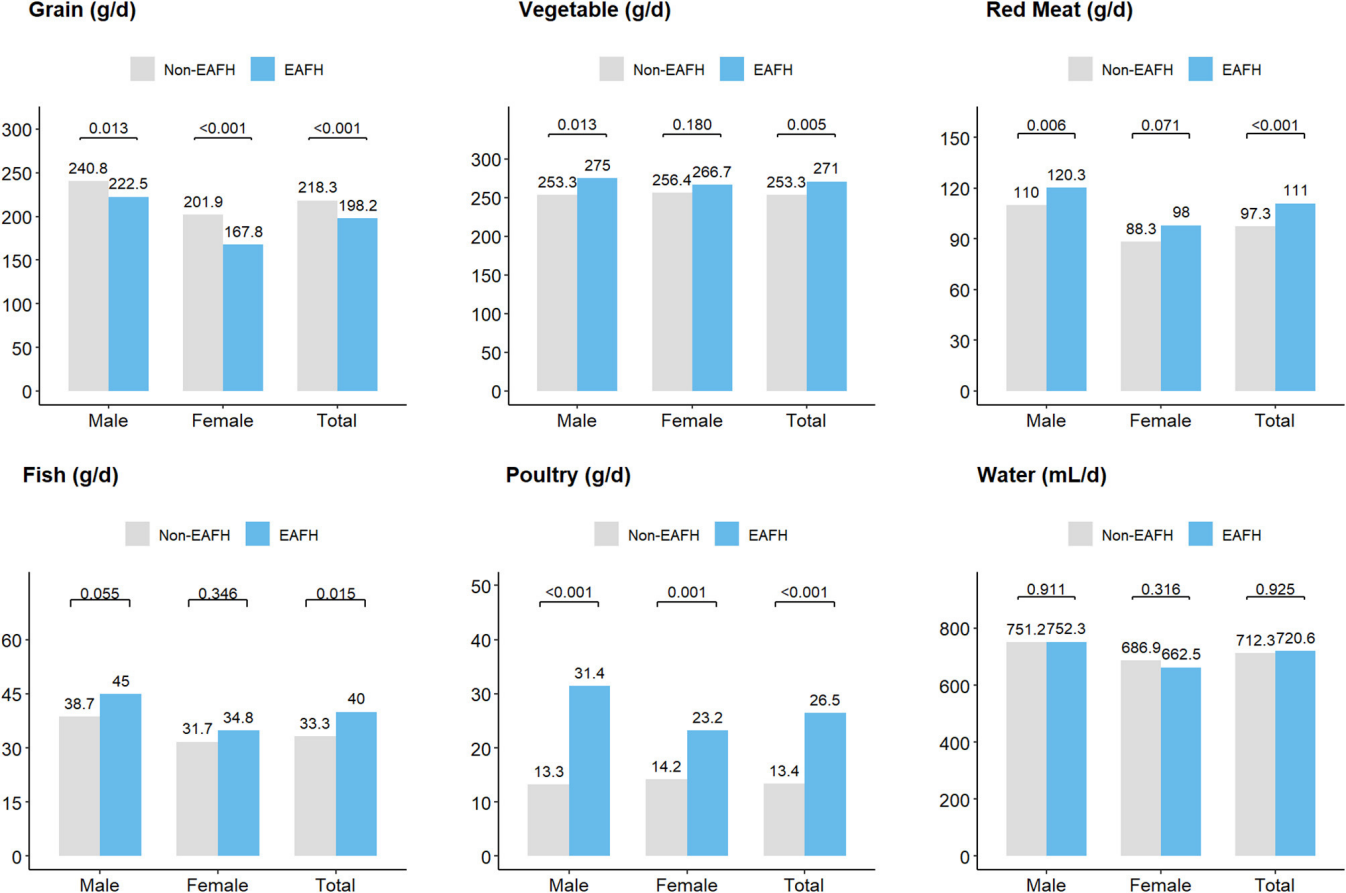
Food intake (g/d) among different populations. EAFH, Eating away from home.

However, with respect to the women, there was a lack of significant difference in red meat, vegetable, fish, and water intake between participants with EAFH and non-EAFH (Figure 3).

**Figure 4:**
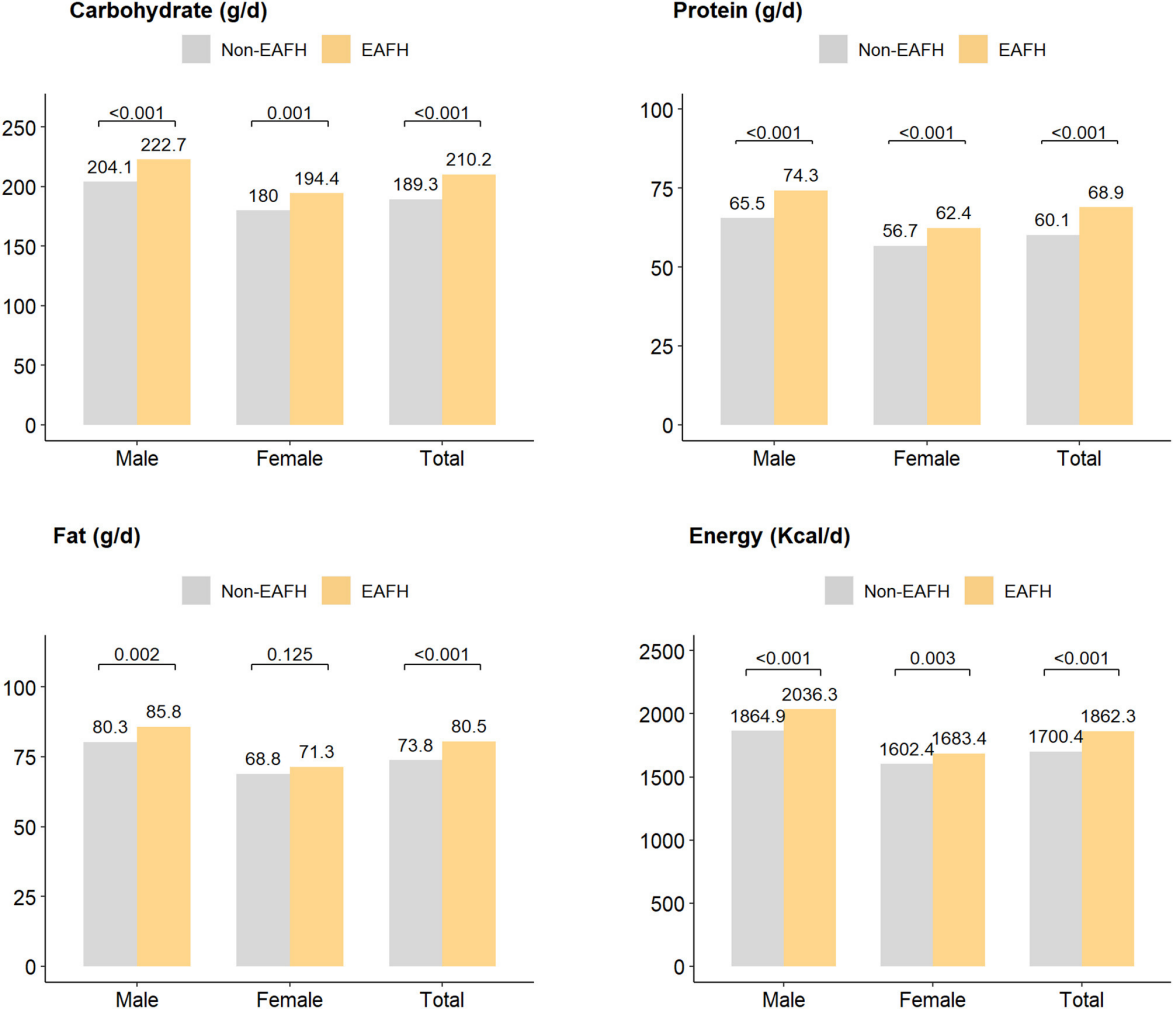
Three major nutrients intake (g/d) and energy intake (Kcal/d) among different populations. EAFH, Eating away from home.

In addition, participants with EAFH had more intake of three major nutrients and energy than those with non-EAFH (Figure 4), and men participants had similar results. However, there was a lack of statistical significance in fat intake between women with EAFH and non-EAFH.

We also compared intake of food, nutrients, and energy between EAFH and non-EAFH regarding different subgroups, and similar results remained (see the Supplementary Tables 1, 2).

## Discussion

The current study was based on a sample size of 3,489 individuals in South China and suggested a 1.27-fold OR of high serum uric acid in adults who had a habit of EAFH, compared with those without EAFH. We also found sex differences regarding the association. A positive association was found in men, but not in women. Adults eating out during breakfast or at a restaurant were inclined to be associated with an increased OR of high serum uric acid. Our findings were robust since the results from subgroup analysis did not have large fluctuations even after adjusting for a broad of potential confounders.

The rate of EAFH has been rising in China in recent years. A previous study demonstrated that the EAFH rate rose from 9.4 to 13.95% between 2004 and 2011 (28). Our study identified an EAFH frequency of 19.9% in 2015, indicative of an upward trend. However, some other places in China, such as Shanghai, reported a higher rate of 55.05% (17). It should be noted that the definition of EAFH varies in these studies. The study by Zeng et al. (28) focused on the place where people take food but did not decompose consumption behaviors by cooking place further, while a study from Shanghai included meals prepared away from home, such as takeaway food but eating at home (17). We restricted EAFH to eating at restaurants or company/school canteen but not takeaway food eating at home. The definitions with some minor differences would be affected the frequencies of eating away from different studies. The inconsistent definitions of EAFH existing in different studies indicated that an accordant one should be proposed.

Previous studies have shown an elevated risk of diet-related diseases and EAFH (14–16). The positive association between EAFH and obesity or an increased BMI has been confirmed by large sample studies (16, 28, 29). Our study discovered an elevated risk of high serum uric acid in adults with EAFH. EAFH has been linked to the consumption of low-quality, high-calorie foods as well as some unhealthy food, such as snacks, beverages, foods in fat and sugar (14, 30, 31). Poor food quality may explain the association between EAFH and high serum uric acid. Our study found that people with EAFH had higher red meat, fat, and total energy intake, especially in restaurants. A previous study has also reported that a high level of surplus energy was associated with the risk of diet-related disease (28). However, how the dietary behaviors exert roles on high serum uric acid should be further study.

Sex discrepancies in the association between EAFH and the OR of high serum uric acid were identified. A positive association was found in men but not in women. Possible reasons are listed as follows. To start with, men are more inclined to have EAFH than women. The rate of EAFH in men (23.6%) was higher than in women (16.9%) in our study. This may be explained by sex differences in family responsibilities. In China, most women should take the responsibility of taking care of the family, which would reduce the frequency of EAFH (15, 28). Furthermore, the diet behavior may induce more impacts on the men than women, which were largely attributable to the differences in amphoteric physiological anatomy and hormone (32, 33). Our study also found a higher proportion of high serum uric acid in men (32.5%) than women (21.2%). In addition, women are more likely to control their body weight by reducing food intake (34, 35), even when eating away from home. Higher food consumption was observed in men with EAFH than those eating at home, while there was a lack of such large differences of food intake (red meat, vegetable, and fish) between women with EAFH and those eating at home in our study.

Several limitations in this study should be discussed. Firstly, although this study was based on a large sample size of 3,489 individuals, the extrapolated conclusion should limit to the adult as only participants over 18 years old were eligible. High serum uric acid is more prevalent in middle-aged adults than young-aged adults or children and adolescents. We did not analyze the different effects regarding various age groups for spatial confined. Secondly, it should be noted that a cross-sectional study cannot draw a causal relationship because of the indeterminately chronological order of the relation and potential confounding factors. We employed a generalized linear mixed-effects model and put possible risk factors into the model as we could to adjust their potential confounding effects. However, effects from other possible risk factors such as family history and co-existing diseases, cannot be eliminated. Thirdly, we did not analyze the effect of consuming takeaway food on high serum uric acid, which might introduce potential misclassification bias. As the rate of consuming takeaway food is increasingly rising, its impact on diet-related disease should be uncovered and further studies are warranted. Fourthly, serum uric acid would be affected by dietary factors with a medium to a long period, while we only applied 3 consecutive 24-h recalls collecting food consumption, which only provided clues to the cause of high serum uric acid. The association between medium or long-term EAHF and the OR of high serum uric acid should be verified.

## Conclusions

This study demonstrated an increased OR of high serum uric acid in adults with EAFH in 3,489 individuals who participated in Guangdong NHS in South China. Sex differences in the association were observed. Compared with those without EAFH, a 1.66-fold OR of high serum uric acid was found in men with EAFH, while the association was not statistically significant in women. We also found sex differences in food intake between EAFH and non-EAFH. Diet behavior has a close link to diet-related diseases and good diet behaviors should be publicly advocated to promote their health.

## Data Availability Statement

The original contributions presented in the study are included in the article/Supplementary Material, further inquiries can be directed to the corresponding author/s.

## Ethics Statement

The studies involving human participants were reviewed and approved by the Ethical Committee of Chinese Center for Disease Control and Prevention. The patients/participants provided their written informed consent to participate in this study.

## Author Contributions

J-wP and Z-hC conceived and designed the experiments. S-wC and PW performed the analyses. S-wC wrote the first draft of the manuscript. PW, G-yJ, QJ, X-mH, W-jM, and RH significantly contributed to the development of the final draft of the manuscript. All authors approve of the final version of the manuscript.

## Funding

This study was supported by Guangdong key research and development program (Grant Nos.: 2019B020210001, 2019B020230001, and 2019B020210002) and Guangdong mandatory medical research fund project (Grant No.: C2019050).

## Conflict of Interest

The authors declare that the research was conducted in the absence of any commercial or financial relationships that could be construed as a potential conflict of interest.

## Publisher's Note

All claims expressed in this article are solely those of the authors and do not necessarily represent those of their affiliated organizations, or those of the publisher, the editors and the reviewers. Any product that may be evaluated in this article, or claim that may be made by its manufacturer, is not guaranteed or endorsed by the publisher.
