# Supplementary material for: Sex Difference in the Association Between Eating Away From Home and the Risk of High Serum Uric Acid in South China
Source: Front Nutr. 2021 Oct 18;8:647287. doi: 10.3389/fnut.2021.647287 (PMC8558310; doi:10.3389/fnut.2021.647287)
Supplement: Supplementary file 1 [file Table_1.docx]

Table s1 Comparison of food intake between Non-EAFH and EAFH regarding breakfast, lunch and dinner.

|  | **Male** | | **P-value** | **Female** | | **P-value** | **Total** | | **P-value** |
| --- | --- | --- | --- | --- | --- | --- | --- | --- | --- |
|  | **Non-EAFH** | **EAFH** |  | **Non-EAFH** | **EAFH** |  | **Non-EAFH** | **EAFH** |  |
| Breakfast |  |  |  |  |  |  |  |  |  |
| n | 1397 | 206 |  | 1715 | 171 |  | 3112 | 377 |  |
| Grain (g/d) | 240(177.3, 315.2) | 218.1(161.7, 289.9) | 0.032 | 198.5(147.8, 265.2) | 170(125.8, 220.1) | <0.001 | 216.7(157.8, 286.7) | 198(147.9, 264) | 0.001 |
| Vegetable (g/d) | 253.3(185, 346.2) | 284.2(216.7, 366) | 0.004 | 256.7(183.3, 340) | 270(196.7, 386.7) | 0.061 | 254.2(183.3, 342) | 280(206.3, 370) | 0.001 |
| Red meat (g/d) | 113.3(66.7, 169.9) | 125.8(83.3, 183.3) | 0.013 | 89.3(50.4, 136.7) | 102(63.3, 141.1) | 0.033 | 100(58.8, 150.3) | 115.8(73.3, 158) | <0.001 |
| Fish (g/d) | 38.7(0, 91.8) | 48.5(19.3, 90.5) | 0.036 | 32.4(0, 75) | 38(16.7, 76.3) | 0.065 | 33.6(0, 82.5) | 43.3(16.7, 83.3) | 0.002 |
| Poultry (g/d) | 16.7(0, 50) | 31.6(0, 66.7) | <0.001 | 15.9(0, 44) | 23.3(0, 46) | 0.060 | 16.7(0, 48.3) | 26.4(0, 52.3) | <0.001 |
| Water (g/d) | 751.2(572.1, 966.8) | 752.3(616.1, 916.4) | 0.792 | 681(522.4, 865.2) | 722.2(553.1, 905) | 0.135 | 710.6(544.6, 913.5) | 739.3(589.5, 905) | 0.080 |
| Carbohydrate (g/d) | 207.6(160.8, 263.7) | 221.7(175.7, 273.2) | 0.016 | 180.7(144.4, 228.8) | 197(160.7, 246.8) | 0.002 | 191.3(150.6, 246) | 212.2(168.8, 262.3) | <0.001 |
| Protein (g/d) | 66.4(52.3, 82.2) | 75.7(62.2, 94.7) | <0.001 | 57.2(45.5, 70.8) | 64.8(51.5, 79.1) | <0.001 | 60.7(47.9, 76.1) | 70(55.9, 89.5) | <0.001 |
| Fat (g/d) | 80.8(59.3, 109.4) | 86.9(67.2, 118.6) | 0.003 | 68.9(51.3, 91.9) | 71.2(55.3, 95.3) | 0.275 | 74.2(54.2, 99.3) | 80.1(62.1, 104.7) | <0.001 |
| Total energy intake (Kcal/d) | 1883.3(1507.7, 2273.4) | 2084.7(1664.6, 2517.1) | <0.001 | 1607(1315.2, 1934) | 1685.5(1411.6, 2023.3) | 0.015 | 1720.8(1380.1, 2106) | 1868.8(1542.5, 2347.1) | <0.001 |
| Lunch |  |  |  |  |  |  |  |  |  |
| n | 1343 | 260 |  | 1657 | 229 |  | 3000 | 489 |  |
| Grain (g/d) | 239.3(176.8, 315.7) | 226.7(161.4, 300.4) | 0.050 | 200(149.2, 266.2) | 161.3(119.7, 222.5) | <0.001 | 217(159.1, 287.2) | 192.9(142.3, 266.7) | <0.001 |
| Vegetable (g/d) | 256.7(190, 346.7) | 265(190.8, 358.1) | 0.513 | 256.7(183.3, 343.3) | 266.7(186.7, 340) | 0.447 | 256.7(185.2, 343.7) | 266.7(190, 353.3) | 0.297 |
| Red meat (g/d) | 113.3(66.7, 170) | 117.8(76.7, 171.7) | 0.091 | 90(50, 138) | 94.9(61.7, 137.3) | 0.389 | 100(58.3, 152.3) | 107.7(70, 153.3) | 0.019 |
| Fish (g/d) | 39.7(0, 93.5) | 42.6(15.5, 83.3) | 0.446 | 33.3(0, 76) | 33.3(6.7, 66.7) | 0.963 | 34.6(0, 83.3) | 37.3(10.5, 75.7) | 0.388 |
| Poultry (g/d) | 16.7(0, 50) | 28.8(0, 69.3) | <0.001 | 15.4(0, 44) | 22(0, 43.3) | 0.023 | 16.7(0, 46.7) | 24.3(0, 55.3) | <0.001 |
| Water (g/d) | 751.2(574.2, 959.3) | 751.6(590.1, 948.3) | 0.619 | 687.3(533, 868.7) | 659(504.3, 856.9) | 0.213 | 714(549.9, 913.5) | 712.8(550.5, 911.2) | 0.966 |
| Carbohydrate (g/d) | 206.1(159.2, 262.9) | 224.7(180.1, 282.6) | 0.001 | 180.2(144.8, 227.8) | 194.2(156.8, 246.2) | 0.005 | 190.8(150.5, 244.5) | 210.4(167.8, 263.1) | <0.001 |
| Protein (g/d) | 66.2(52.4, 81.7) | 74.4(60.4, 91.8) | <0.001 | 57.3(45.5, 70.8) | 60.8(50, 75.9) | 0.001 | 60.7(48, 76.1) | 68.4(53.2, 86.1) | <0.001 |
| Fat (g/d) | 80.7(59.6, 109.4) | 86.8(64.7, 114.7) | 0.031 | 69.1(51.2, 92.2) | 69.2(54, 90.4) | 0.563 | 74.6(54.3, 99.6) | 80.1(58.6, 102.6) | 0.010 |
| Total energy intake (Kcal/d) | 1881.7(1498.9, 2282.1) | 2007.3(1671.5, 2491) | <0.001 | 1608.3(1316.3, 1933) | 1677.7(1370, 2023.3) | 0.053 | 1715.6(1378.9, 2106.2) | 1856.8(1520.1, 2283.5) | <0.001 |
| Dinner |  |  |  |  |  |  |  |  |  |
| n | 1484 | 119 |  | 1797 | 89 |  | 3281 | 208 |  |
| Grain (g/d) | 239.8(176.6, 315.1) | 215.1(160.1, 272.5) | 0.015 | 198(147.8, 263.4) | 159.1(113.8, 205.4) | <0.001 | 216.7(157, 286.7) | 184.7(137, 249.9) | <0.001 |
| Vegetable (g/d) | 256.7(190, 346.7) | 266.7(186.7, 366.7) | 0.505 | 256.7(183.5, 341.7) | 280(180, 383.3) | 0.337 | 256.7(186.7, 343.3) | 273.3(181.7, 370) | 0.243 |
| Red meat (g/d) | 115(66.7, 171.8) | 115.5(73.3, 166.7) | 0.660 | 90(51.7, 137.3) | 93.8(56.7, 140) | 0.599 | 100(60, 152.7) | 108.6(64.7, 153.7) | 0.210 |
| Fish (g/d) | 39.3(0, 90.3) | 50(19.3, 96.7) | 0.082 | 33.3(0, 75.7) | 28(5, 63.3) | 0.517 | 35(0, 82.5) | 38.8(12.8, 83.3) | 0.238 |
| Poultry (g/d) | 17.6(0, 50) | 34(0, 70.4) | 0.001 | 16.7(0, 44) | 22.7(0, 39.6) | 0.223 | 16.7(0, 48.3) | 30.1(0, 63.7) | <0.001 |
| Water (g/d) | 750.9(576.4, 956.2) | 771.9(589.5, 1006.7) | 0.178 | 684.5(529, 866.3) | 663.7(523.7, 881.7) | 0.969 | 712.9(548.2, 908.4) | 727.6(577.5, 955.9) | 0.170 |
| Carbohydrate (g/d) | 208.4(161.7, 264.1) | 220.8(175.7, 284.8) | 0.094 | 181.7(145.1, 230.6) | 191.8(157.5, 228.4) | 0.314 | 192.5(151.9, 246.8) | 206.8(170.9, 261.8) | 0.014 |
| Protein (g/d) | 67(52.7, 82.8) | 73.8(61, 93.2) | <0.001 | 57.6(45.9, 71.1) | 62(50.2, 78.4) | 0.027 | 61.2(48.3, 76.9) | 70.2(54.7, 87.9) | <0.001 |
| Fat (g/d) | 81.9(60.2, 109.4) | 82.8(62.1, 118.9) | 0.265 | 69.1(51.3, 91.9) | 74.6(55.2, 92.5) | 0.093 | 74.8(54.6, 99.6) | 81.2(61.2, 106.5) | 0.013 |
| Total energy intake (Kcal/d) | 1890.3(1524.7, 2297.9) | 2056.7(1610.2, 2527.9) | 0.003 | 1613.1(1322.3, 1940.9) | 1648(1411.6, 2040) | 0.162 | 1734.4(1391.2, 2120.3) | 1875.5(1501.8, 2361.2) | <0.001 |

Footprint：EAFH=eating away from home.

Table s2 Comparison of food intake between Non-EAFH and EAFH regarding restaurant and canteen.

|  | **Male** | | **P-value** | **Female** | | **P-value** | **Total** | | **P-value** |
| --- | --- | --- | --- | --- | --- | --- | --- | --- | --- |
|  | **Non-EAFH** | **EAFH** |  | **Non-EAFH** | **EAFH** |  | **Non-EAFH** | **EAFH** |  |
| **Restaurant** |  |  |  |  |  |  |  |  |  |
| n | 1327 | 276 |  | 1703 | 183 |  | 3030 | 459 |  |
| Grain (g/d) | 239.5(176.7, 315.3) | 225(165.8, 299.2) | 0.001 | 200(149.2, 265.2) | 160(118.3, 215.8) | 0.110 | 216.7(158.3, 286.2) | 201.9(144.5, 272.2) | <0.001 |
| Vegetable (g/d) | 255(189.3, 346.2) | 271(193.3, 360.5) | 0.050 | 256.7(183.3, 341.7) | 266.7(190, 360) | 0.181 | 256.4(185, 343.3) | 270(191.7, 360) | 0.188 |
| Red meat (g/d) | 113.3(66.7, 169.9) | 121(80.8, 182.6) | <0.001 | 89.3(50.3, 136.7) | 100(61.7, 141.1) | 0.015 | 99.3(58.3, 150) | 114(71.3, 163.3) | 0.053 |
| Fish (g/d) | 38.7(0, 90.2) | 47.4(19.3, 94.7) | <0.001 | 31.7(0, 75.2) | 38(10.5, 73.5) | 0.005 | 33.3(0, 81.7) | 43(16.7, 85) | 0.167 |
| Poultry (g/d) | 16.7(0, 50) | 31.6(0, 67.8) | <0.001 | 15.7(0, 44) | 24.1(0, 50) | <0.001 | 16.7(0, 46.3) | 26.7(0, 56.3) | 0.020 |
| Water (g/d) | 753.2(573.7, 969.1) | 746.9(592.2, 913.8) | 0.406 | 681.9(525.3, 866.8) | 715.8(547.9, 863.6) | 0.572 | 710.7(545.5, 914.7) | 732(578.6, 891.4) | 0.531 |
| Carbohydrate (g/d) | 206.9(159.8, 263.5) | 221.5(175.8, 275.3) | <0.001 | 181.1(144.8, 228.7) | 193.4(156.9, 246.8) | 0.004 | 190.9(150.4, 244.9) | 212.2(167.7, 262.4) | 0.019 |
| Protein (g/d) | 66.3(52, 81.7) | 74.6(60.5, 93.1) | <0.001 | 56.9(45.4, 70.8) | 64.3(52.5, 79.1) | <0.001 | 60.5(47.7, 75.6) | 70(56.5, 89.4) | <0.001 |
| Fat (g/d) | 81(58.9, 109.6) | 84.9(66, 112) | <0.001 | 68.8(51.3, 91.6) | 71.9(54.9, 96.8) | 0.027 | 74(54, 99) | 81.6(62.1, 104.2) | 0.134 |
| Total energy intake (Kcal/d) | 1881.7(1490.9, 2276.4) | 2044.7(1657.3, 2468.4) | <0.001 | 1606.5(1316, 1934.6) | 1680.4(1393.5, 2015.3) | <0.001 | 1713.5(1375.2, 2103.2) | 1895.7(1544, 2361.4) | 0.013 |
| **Canteen** |  |  |  |  |  |  |  |  |  |
| n | 1456 | 147 |  | 1720 | 166 |  | 3176 | 313 |  |
| Grain (g/d) | 239.8(176.9, 315.1) | 215.1(156.1, 298.3) | <0.001 | 198.4(147.4, 265.3) | 169.2(126.7, 222.8) | 0.010 | 216.7(157.9, 287) | 186.9(143.4, 252.3) | <0.001 |
| Vegetable (g/d) | 254.2(186.7, 346.7) | 283.3(216.7, 365) | 0.007 | 256.7(183.3, 341.9) | 275.8(196.7, 351.7) | 0.009 | 256.7(183.3, 343.7) | 280(203.3, 356.7) | 0.230 |
| Red meat (g/d) | 113.3(66.7, 170.7) | 122.3(83.3, 170) | 0.098 | 90(50.5, 138.4) | 95.8(61.7, 136) | 0.075 | 100(59.1, 153.3) | 108.8(72.6, 150) | 0.564 |
| Fish (g/d) | 40(0, 93.4) | 38.7(16.5, 75.7) | 0.835 | 33.3(0, 75.9) | 33.3(5, 66.7) | 0.997 | 35.1(0, 83.3) | 35(11.7, 66.7) | 0.848 |
| Poultry (g/d) | 17.2(0, 50) | 31.7(0, 76.7) | <0.001 | 16.7(0, 44) | 22.3(0, 44) | <0.001 | 16.7(0, 48.3) | 26.4(0, 55) | 0.056 |
| Water (g/d) | 750.5(573.1, 957.4) | 772.7(628.9, 965.9) | 0.883 | 686.9(532.1, 870.6) | 657.3(507.5, 832.9) | 0.150 | 714.5(549.5, 913.2) | 705(553.8, 913) | 0.188 |
| Carbohydrate (g/d) | 207.7(160.9, 264.1) | 225.6(180.2, 282.2) | <0.001 | 180.2(144.9, 229.4) | 196.7(157.5, 240.2) | 0.014 | 191.8(151.7, 246.4) | 210.4(167.7, 259.9) | 0.010 |
| Protein (g/d) | 66.6(52.9, 82.6) | 74.9(61.4, 90.4) | <0.001 | 57.4(45.6, 70.9) | 60.2(48.9, 75.9) | <0.001 | 61.2(48.3, 76.9) | 68.4(52.1, 83.7) | 0.006 |
| Fat (g/d) | 80.9(60.1, 108.7) | 90.9(63.3, 120.5) | 0.056 | 69.2(51.3, 92.3) | 68.6(53.3, 89.3) | 0.016 | 74.8(54.6, 99.6) | 79.5(58.5, 104.5) | 0.715 |
| Total energy intake (Kcal/d) | 1887.7(1517.7, 2296) | 2071.9(1670.5, 2490.3) | <0.001 | 1609.6(1320.1, 1934.3) | 1671.4(1367.9, 2027.3) | <0.001 | 1729.8(1389.9, 2116.5) | 1838.4(1472.2, 2264.7) | 0.114 |

Footprint：EAFH=eating away from home.
